# Dietary approach to stop hypertension and healthy eating index 2015, modify the association between FTO polymorphisms and obesity phenotypes

**DOI:** 10.1186/s13104-023-06463-3

**Published:** 2023-09-11

**Authors:** Firoozeh Hosseini-Esfahani, Mahshid Rezaei, Glareh Koochakpoor, Maryam S. Daneshpour, Parvin Mirmiran, Fereidoun Azizi

**Affiliations:** 1 Nutrition and Endocrine Research Center, Research Institute for Endocrine Sciences, Shahid Beheshti University of Medical Sciences, P.O.Box: 19395-4763, Tehran, Iran; 2 Maragheh University of Medical Sciences, Maragheh, Iran; 3 Cellular and Molecular Endocrine Research Center, Research Institute for Endocrine Sciences, Shahid Beheshti University of Medical Sciences, Tehran, Iran; 4 Endocrine Research Center, Research Institute for Endocrine Sciences, Shahid Beheshti University of Medical Sciences, Tehran, Iran

**Keywords:** Healthy diet, FTO, Obesity, Interaction

## Abstract

This study aimed to investigate the interaction of the healthy eating index (HEI) and the dietary approach to stop hypertension (DASH) diet scores with FTO polymorphisms in relation to change in obesity traits. A total of 4480 subjects aged ≥ 18 years were selected from participants of the Tehran lipid and glucose study and followed-up 3 years. Selected polymorphisms (rs1421085, rs1121980, rs8050136) were genotyped and genetic risk score (GRS) was computed. HEI and DASH scores were computed based on dietary data. Changes in body mass index (BMI), waist circumference (WC), waist to hip ratio (WHR) and visceral adiposity index (VAI) were measured. Higher adherence to both DASH and HEI scores were increased with higher ages. Individuals with high GRS had a lower change in BMI when they had higher adherence to HEI, compared to subjects with lower HEI score (P trend = 0.01). Change in WC in participants in the fourth quartile of HEI score in minor allele carriers of FTO variants was lower compared to the first quartile; conversely, higher adherence to the DASH score by this genotypic group was related to increase in WC. No significant interaction was seen between FTO polymorphisms and both diet scores regarding changes in any of obesity traits. In conclusion, in individuals with high GRS higher adherence to HEI score was associated with lower change in BMI and WC, while higher adherence to DASH diet was associated with higher change in WC, compared to individuals with lower adherence to both scores.

**Supplementary Information:**

The online version contains supplementary material available at 10.1186/s13104-023-06463-3.

## Introduction

Obesity is a condition in which excess fat accumulates in the body and could adversely affect one’s health [1]. According to the latest report conducted by the world health organization, about 1.9 billion people are overweight, of whom 600 million suffer from obesity [2]. It is also regarded as a major risk factor for several chronic diseases such as coronary heart disease, musculoskeletal disorders, and some types of cancers [3].

Mainstream medicine views obesity as a result of chronic energy imbalance. In recent decades, changes in dietary and physical activity patterns have contributed to the increased rate of obesity [4], affirming the role of the environment [5]; however, in any given environment, there is considerable individual variation in body weight and fat mass, suggesting that weight is influenced by complex interactions between genetic and environmental factors [6]. This speculation has been confirmed by numerous studies investigating the matter in various situations [7].

Advances in genetic technology have helped us to identify several common genetic variants associated with obesity. The most commonly implicated gene is fat mass and obesity associated gene (FTO) [8]. There are several single nucleotide polymorphisms (SNPs) on FTO that has been associated with obesity in different populations [9].

Of lifestyle factors affecting body composition, diet is of utmost importance. Dietary approaches to stop hypertension (DASH) which is advised for preventing and treating high blood pressure, has been attributed to lower risk of cardiovascular diseases (CVD), cancer, insulin resistance, and blood lipid markers [10]. Healthy eating index (HEI) is a measure of diet quality which was seen to have inverse relationships with serum levels of cholesterol, C-reactive protein, homocysteine, glucose and HbA1c [11]. Studies confirming the contribution of these indices (posteriori dietary patterns) to modifying obesity phenotypes are in abundance [12, 13]. On the other hand, available research indicated that there were certain genetic variants such as the FTO and MC4R [14, 15], which were associated with adiposity; these variants may influence food preference patterns such as increased intake of sugar and carbohydrate consumption [16], total energy intake and preferences in macronutrient intakes [14, 15]. However, studies investigating the interaction of FTO polymorphisms with posteriori-dietary patterns are scarce especially in the Middle East population. Also the previous contradictory results on the relationship of dietary patterns with obesity may be due to the modifying effect of FTO polymorphisms, so we performed this prospective study to find out the interaction of DASH and HEI scores with FTO polymorphisms in isolation or in a combined form genetic risk score (GRS) concerning change in obesity traits among adult Tehranian participants.

## Methods

### Study population

Subjects of this cohort study were chosen from participants of the Tehran Lipid and Glucose Study (TLGS), a population-based ongoing study performed to determine risk factors for non-communicable diseases in a group of residents of District 13 of Tehran, the capital of Iran. The first survey was done from 1999 to 2001 on 15,005 individuals aged ≥ 3 years, using the multistage stratified cluster random sampling technique, and follow-up surveys were performed every 3 years; 2002–2005 (survey 2), 2005–2008 (survey 3), 2008–2011(survey 4), and 2012–2015 (survey 5) to find out recently non-communicable diseases [17].

Of 12,823 individuals attending the fourth phase of the TLGS (2008–2011) 8843 adults were ≥ 18 years. Subjects were removed due to anthropometric and nutritional missing data (n = 1961), so the data of 6882 subjects entered in this study as the baseline population and were followed-up to the survey 5 (2011–2014). Exclusion criteria were in these ways; subjects who had not DNA samples or lacking DNA purification in the range of 1.7 < A260/A280 < 2 or incomplete follow up data (n = 1600), pregnant (n = 80) or lactating women (n = 85), those with under- or over-reporting of energy intake (< 800 or ≥ 4200 kcal/day) (n = 637). Overall, data provided by 4480 subjects were entered in this study.

### Ethical approval

A written informed consent form was signed by all participants before entering the study. The ethics committee of the Research Institute for Endocrine Sciences, Shahid Beheshti University of Medical Sciences accepted the study protocol. All methods were carried out according to their relevant regulations and guidelines.

### Dietary assessment

A valid and reliable 168-item semi-quantitative food frequency questionnaire (FFQ) was applied for dietary assessment [18]. Skilled nutritionists questioned the intake of food items with standard serving sizes by face-to-face interviews. The frequency intake of each food item was changed to intake per day (gram/day) using local household measures. Since the Iranian food composition table (FCT) is not complete, the United States Department of Agriculture (USDA) FCT was used to analyze nutrients of foods.

The DASH diet primarily developed to prevent hypertension. The DASH index is a posteriori-dietary pattern which calculates diet quality score. The index score (ranging 8–40) was based on eight food groups or nutrients including fruit, vegetable, nuts and legumes, low-fat dairy products, whole grains, sodium, sweetened beverages, red and processed meats intakes. Individual dietary food groups were calculated per 1000 kcal intake for each item, and then were categorized into quintiles. Subjects were given a score of 1 to 5 for each item. Subjects in the highest quintile intake of sodium, red and processed meat, and sweetened beverage were given a score of 0, and those in the lowest quintile of these food items were given a score of five. For fruit, vegetable, whole grain, low fat dairy, nuts and legumes, those in the highest quintile was given a score of 5 [19].

The HEI is based on key recommendations of the 2015–2020 dietary guidelines for Americans which is composed of 13 dietary integrals; nine of them are adequacy components, including total fruits, whole fruits, total vegetables, greens and beans, whole grains, dairy, total protein foods, seafood and plant proteins, and fatty acids. Four of them are moderation components (those that should be limited) including refined grain, sodium, added sugars, and saturated fats. The HEI score is depend on density, so the amount per 1000 kcal of food groups and ratio of fatty acids was calculated. Recommendations are in the range of 1200–2400 kcal. Six components of adequacy components, including total fruit, whole fruit, total vegetables, greens and beans, total protein foods, seafood and plant proteins, each acquired a score of 0 and 5 for the lowest and highest intake, respectively. The other three adequacy components (whole grains, dairy and fatty acids) were scored from 0 to 10 for the lowest and highest intake, respectively. The four moderation components including refined grains, sodium, added sugars, and saturated fats (SFA), gave a score of 0 and 10, respectively for the highest and lowest intakes. The score of intermediate intakes (between the minimum and maximum) were estimated. The sum of 13 integral scores was computed for a total HEI score ranging from 0 to 100. Individuals with a higher total HEI score had greater adherence to dietary guideline recommendations [20]. The DASH and HEI scores were considered as independent variables in this study.

### Anthropometric measurements

Trained technician measured participants’ body weight with minimal clothing using a calibrated digital scale (model 707, Seca, Hamburg, Germany) and rounded to the nearest 0.1 kg. They measured heights (cm) by a stadiometer (model 208 Portable Body Meter Measuring Device; Seca) and rounded to the nearest 0.5 cm, while the subjects standing without shoes in a normal position. Waist circumference (WC) was measured over light clothing, without exerting any pressure on the umbilicus using an un-stretched tape meter. Hip circumference was assessed at the level of maximal protrusion of the gluteal muscles. By dividing WC (cm) to hip circumference (cm), the waist to hip ratio (WHR) was calculated.

VAI was estimated by the following formulas for men and women separately [21].

$$\text{M}\text{a}\text{l}\text{e}\text{s}: \text{V}\text{A}\text{I}=\left(\frac{\text{W}\text{C}}{39.68+\left(1.88\times \text{B}\text{M}\text{I}\right)}\right)\times \left(\frac{\text{T}\text{G}}{1.03}\right)\times \left(\frac{1.31}{\text{H}\text{D}\text{L}}\right)$$

$$\text{F}\text{e}\text{m}\text{a}\text{l}\text{e}\text{s}: \text{V}\text{A}\text{I}= \left(\frac{\text{W}\text{C}}{36.58+\left(1.89\times \text{B}\text{M}\text{I}\right)}\right) \times \left(\frac{\text{T}\text{G}}{0.81}\right)\times \left(\frac{1.52}{\text{H}\text{D}\text{L}}\right)$$

Anthropometric changes were considered as the outcomes or dependent variables in this study. BMI change was calculated by subtracting the BMIs obtained at the following survey from its corresponding at the baseline. The increase or decrease in BMI was determined if the BMI change was > 0 or ≤ 0 respectively. WHR, WC, and VAI change was computed using the same principle.

### Physical activity assessment

Trained interviewer questioned physical activity with the Persian-translated modifiable activity questionnaire (MAQ). MAQ has high reliability and moderate validity. Data on the time and frequency of light, moderate, hard and very hard intensity typical activities over the last year were collected. The level of physical activity levels were estimated based on metabolic equivalent (MET)-hours/week (MET/h/week) [22].

### Genotype

The region of the FTO gene was evaluated based on the Phenotype-Genotype Integrator and the authenticated catalog of published genome-wide related studies. FTO SNPs were chosen based on the available data, minor allele frequency > 0.2, and P values < 10^− 7^. Six FTO single nucleotide polymorphisms (SNPs) were selected which were related to both dietary patterns and obesity phenotypes, including rs1421085, rs1121980, rs17817449, rs8050136, rs9939973, and rs3751812. There were a strong correlation between rs8050136, rs1421085, and rs1121980 (r^2^ > 0.8) with the other three SNPs in our analyses, so we applied these SNPs in our study. Also, there were a moderate relationship (r^2^ < 0.7) between these three SNPs [23].

Genomic DNA was derived from non-coagulated whole blood samples, using a standard proteinase K, salting-out method. The evaluation of DNA quality was done by a Thermo Scientific NanoDrop 1000 Spectrophotometer. DNA purification outside the range of 1.7 < A260/A280 < 2 were excluded due to low quality. Extracted DNAs were aliquoted into 1.5-ml tubes and kept in ultra-freezers at -80 C, for preservation. HumanOmniExpress-24-v1-0 bead chips, containing 649,932 SNP loci was used to genotype the portions of DNA samples according to the manufacturer’s qualifications (Illumina Inc., San Diego, CA, USA), an average mean distance of 4 kb at the deCODE genetics company (Reykjavik, Iceland) was regarded. PLINK program (V 1.07) and R statistic (V 3.2) were used to evaluate the quality, with a total genotyping rate of 0.9774. Finally, genotyping data of FTO SNPs (rs8050136, rs1421085, and rs1121980) were analyzed [24].

### Obesity GRS calculation

The weighted method was applied for GRS calculation based on 3 selected SNPs. Based on the existence of risk alleles (BMI increasing allele), the coefficients of 0, 1, and 2 were allocated to each SNP. The weight of each SNP was obtained from the logistic regression analysis performed on the study population. GRS score is calculated by multiplying the coefficient of its risk alleles with the weight of each SNP.

GRS = (OR1 × SNP1 + OR2 × SNP2 + OR3 × SNP3) × (n/sum of the ORs).

OR is the odds ratio of each individual SNP on BMI. The logistic regression analysis was used to determine coefficients for the standardized weighted GRS in our population. The coefficients for risk alleles of rs1121980, rs1421085 and rs8050136 were respectively 0.21, 0.23, 0.24. Three SNPs (dominant model) were considered as independent variables and obesity (BMI ≥ 30 and BMI < 30) as dependent variable [25].

### Statistical analysis

Data were analyzed using SPSS version 21, and the statistical significance was considered as P < 0.05. To evaluate the qualitative and quantitative variables across quartiles of DASH and HEI scores, Chi-square and ANOVA tests were used respectively. The Pearson’s Chi-square test was applied to test The Hardy-Weinberg equilibrium.

The interaction of diet quality scores with FTO gene variants (dominant model) or GRS concerning WHR, WC, VAI, and BMI alteration was estimated using a general linear model (ANCOVA). Participants were categorized into 8 groups based on three SNP polymorphisms (dominant model) and quartiles of dietary scores (HEI and DASH) to estimate mean ± SEM changes of obesity phenotypes. Participants were categorized into two groups based on the median of GRS (> 6.81 and ≤ 6.81). General linear models were performed to estimate the interactions of GRS with quartiles of DASH and HEI score concerning changes of WHR, WC, VAI, and BMI.

The potential confounders, including smoking status (current, ex-smoker, or never smoked), age, gender, physical activity (low, moderate, and high), education levels (> 14 and ≤ 14 years), and energy intake were taken into account in all models.

## Results

Baseline characteristics of participants across quartiles of HEI and DASH scores are presented in Table 1. Individuals with higher adherence to DASH and HEI score were particularly older and smoked less (P < 0.001). However, higher WC at baseline was associated with more adherences to both DASH and HEI scores. Baseline BMI was higher in the fourth quartile of DASH score, and baseline VAI was higher in the fourth quartile of HEI score of participants. Although changes in WC were inversely associated with HEI score of participants, no significant relationship had been indicated between changes in other anthropometric measures such as BMI, waist/hip ratio, VAI and DASH or HEI scores among individuals.

**Table 1:** Characteristics of the study population according to posteriori- dietary patterns among adult participants of the Tehran Lipid and Glucose Study (n = 4480)

Characteristics	Quartiles of HEI score					Quartiles of DASH score				
	Q1 < 60.18	Q2 < 60.16- ≤ 65.19	Q3 < 65.20- ≤ 70.22	Q4 ≤ 70.23	P	Q1 < 20	Q2 21< - ≤ 24	Q3 25<- ≤ 27	Q4 ≤ 28	P
**Mean**	55.3 ± 4.2	62.8 ± 1.4	67.6 ± 1.5	74.0 ± 3.13	< 0.001	17.8	22.6	25.9	30.4	< 0.001
**Age**	38.3 ± 13.2	39.2 ± 13.5	40.4 ± 13.8	43.8 ± 14.3	< 0.001	36.4 ± 13.1	39.6 ± 13.4	41.7 ± 13.6	44.9 ± 14.1	< 0.001
**Current smokers (%)**	10.4%	10.3%	0.7%	8.7%	0.02	10.5%	9.6%	9.5%	6.8%	0.03
**Education level** **≥ 14 years (%)**	19.1%	20.2%	20.3%	22.2%	0.36	18.7%	20.0%	23.3%	20.5%	0.07
**Men (%)**	40.9%	44.1%	47.3%	48.9%	< 0.001	58.7%	44.1%	41.2%	35.3%	< 0.001
**Physical activity (MET/min/week)**	332 ± 428	344 ± 381	820 ± 1121	445 ± 470	0.13	439 ± 589	403 ± 493	436 ± 579	770 ± 1173	0.55
**Baseline BMI (Kg/m** ^**2**^ **)**	26.7 ± 4.80	27.1 ± 4.77	27.1 ± 4.71	27.7 ± 4.63	1.00	26.7 ± 4.80	27.1 ± 4.77	27.1 ± 4.70	27.7 ± 4.63	< 0.001
**Changes in BMI (Kg/m** ^**2**^ **)**	0.48 ± 2.05	0.38 ± 1.84	0.41 ± 2.43	0.33 ± 1.80	0.39	0.48 ± 2.05	0.38 ± 1.84	0.42 ± 2.42	0.33 ± 1.80	0.45
**Baseline WC (cm)**	90.1 ± 12.5	91.1 ± 12.0	92.1 ± 11.9	94.01 ± 12.0	< 0.001	91.8 ± 12.7	91.5 ± 12.7	91.6 ± 11.9	92.5 ± 11.7	0.22
**Change in WC (cm)**	0.70 ± 6.14	0.92 ± 6.03	0.63 ± 6.34	0.06 ± 5.83	0.01	0.92 ± 6.01	0.67 ± 6.04	0.38 ± 6.15	0.24 ± 6.18	0.07
**Baseline waist/hip ratio**	0.90 ± 0.08	0.91 ± 0.08	0.92 ± 0.08	0.93 ± 0.08	< 0.001	0.92 ± 0.08	0.91 ± 0.08	0.92 ± 0.08	0.91 ± 0.08	0.22
**Change in waist/hip ratio**	0.01 ± 0.05	0.01 ± 0.23	0.01 ± 0.05	0.01 ± 0.20	0.64	0.01 ± 0.04	0.01 ± 0.05	0.02 ± 0.31	0.01 ± 0.15	0.21
**Baseline VAI (Kg)**	2.01 ± 1.44	2.37 ± 2.07	2.37 ± 2.07	2.37 ± 1.78	< 0.001	2.19 ± 1.64	2.29 ± 1.85	2.22 ± 1.73	2.36 ± 2.01	0.10
**Change in VAI (Kg)**	-0.01 ± 0.12	-0.00 ± 0.16	-0.01 ± 0.14	0.02 ± 0.14	0.15	-0.01 ± 0.15	-0.01 ± 0.15	-0.01 ± 0.13	-0.01 ± 0.13	0.86

BMI: Body mass index, WC: waist circumference, MET: Metabolic Equivalent, VAI: Visceral Adiposity index, HEI: Healthy Eating Index, DASH: Dietary approach to stop hypertension

^1^Continuous variables were reported as mean ± SD (using ANOVA test)

^2^Categorical variables were analyzed using the Chi-square test and reported as a percentage (%)

There was no statistically significant deviation from the Hardy–Weinberg equilibrium for the three polymorphisms. The median of GRS among participants was 6.81.

Interactions of HEI and DASH scores by FTO SNPs (rs1121980, rs1421085, rs8050136) and GRS concerning changes in BMI and WHR were described in Table 2. HEI modulates the association between FTO SNPs and changes in BMI. Individuals with minor allele (risk allele) carriers of rs1121980 had a lower change in BMI when they had higher adherence to HEI, compared to subjects with lower HEI score (P trend = 0.01). In subjects with wild type homozygote genotype, no significant relationship was observed between HEI score and BMI change (P interaction or Pi = 0.35). Furthermore, in wild type homozygote genotype of rs8050136 and rs1421085, greater adherence to HEI showed a lower change in BMI (P trend = 0.02). A significant trend was found between GRS ≥ 6.81 and HEI score concerning changes in BMI (P trend = 0.01). Adherence to the DASH diet after 3 years of follow up was significantly associated with higher changes in WHR for minor allele carriers of FTO SNPs (rs1121980, rs1421085, rs8050136) and in individuals with GRS ≥ 6.81. There were no interactions between FTO SNPs, GRS and HEI or DASH scores in relation to changes in BMI or WHR.

**Table 2:** Changes in BMI and WHR, according to quartiles of HEI and DASH score by FTO genotypes and GRS in adult participants of the Tehran Lipid and Glucose Study

	BMI ^a^									WHR ^b^								
	HEI score																	
	Q1	Q2	Q3			Q4	P trend	Pi^c^		Q1	Q2		Q3	Q4		P trend	Pi^c^	
**rs1121980**								0.35									0.60	
CC	0.45 + 1.94	0.46 + 1.8	0.37 + 2.0			0.27 + 2.18	0.49			0.01 + 0.05	0.020.25		0.01 + 0.05	0.01 + 0.21		0.08		
CT + TT	0.45 + 1.92	0.52 + 2.8	0.50 + 1.9			0.37 + 1.91	0.01			0.00 + 0.04	0.01 + 0.06		0.01 + 0.05	0.00 + 0.05		0.33		
**rs1421085**								0.68									0.64	
TT	0.44 + 2.29	0.46 + 1.77	0.37 + 2.04			0.15 + 1.60	0.02			0.00 + 0.01	0.01 + 0.05		0.01 + 0.05	0.00 + 0.03		0.81		
TC + CC	0.46 + 1.69	0.48 + 2.11	0.42 + 2.02			0.38 + 2.40	0.33			0.01 + 0.05	0.02 + 0.36		0.01 + 0.05	0.02 + 0.31		0.11		
**rs8050136**								0.53									0.59	
GG	0.45 + 2.22	0.49 + 1.78	0.38 + 1.97			0.16 + 1.61	0.02			0.01 + 0.06	0.02 + 0.35		0.01 + 0.05	0.02 + 0.30		0.86		
GA + AA	0.45 + 1.71	0.46 + 2.12	0.41 + 2.02			0.38 + 2.44	0.19			0.00 + 0.05	0.01 + 0.05		0.01 + 0.05	0.00 + 0.05		0.13		
**GRS**								0.68									0.12	
GRS < 6.81	0.45 + 1.85	0.64 + 2.77	0.47 + 2.06			0.33 + 1.93	0.76			0.00 + 0.04	0.01 + 0.05		0.01 + 0.05	0.00 + 0.06		0.42		
GRS ≥ 6.81	0.45 + 1.94	0.44 + 1.78	0.38 + 2.02			0.27 + 2.02	0.01			0.01 + 0.05	0.02 + 0.25		0.01 + 0.05	0.01 + 0.21		0.36		
			**DASH score**															
**rs1121980**								0.13									0.40	
CC	0.43 + 2.35	0.42 + 1.87	0.80 + 2.5			0.19 + 1.95	0.48		0.01 + 0.4		0.00 + 0.6	0.01 + 0.5		0.01 + 0.4	0.12			
CT + TT	0.49 + 1.99	0.37 + 1.84	0.33 + 2.4			0.34 + 1.75	0.10		0.01 + 0.05		0.01 + 0.05	0.01 + 0.05		0.02 + 0.33	0.01			
**rs1421085**								0.42									0.78	
TT	0.55 + 2.07	0.45 + 1.82	0.40 + 2.6			0.32 + 1.76	0.12		0.01 + 0.4		0.00 + 0.4	0.00 + 0.5		0.00 + 0.5	0.11			
TC + CC	0.37 + 2.02	0.28 + 1.89	0.43 + 2.0			0.36 + 1.83	0.61		0.01 + 0.6		0.01 + 0.4	0.01 + 0.4		0.04 + 0.1	0.01			
**rs8050136**								0.35									0.74	
GG	0.55 + 2.07	0.45 + 1.82	0.40 + 2.6			0.32 + 1.76	0.29		0.01 + 0.4		0.00 + 0.5	0.00 + 0.5		0.00 + 0.5	0.12			
GA + AA	0.43 + 2.01	0.31 + 1.88	0.41 + 1.9			0.34 + 1.80	0.31		0.01 + 0.5		0.01 + 05	0.01 + 0.5		0.04 + 0.8	0.01			
**GRS**								0.13									0.40	
GRS < 6.81	0.46 + 2.28	0.45 + 1.98	0.74 + 2.1			0.26 + 2.05	0.60		0.01 + 0.4		0.00 + 0.5	0.00 + 0.5		0.01 + 0.5	0.19			
GRS ≥ 6.81	0.49 + 1.99	0.37 + 1.81	0.33 + 2.3			0.34 + 1.75	0.15		0.01 + 0.5		0.01 + 0.5	0.01 + 0.5		0.02 + 0.3	0.01			

HEI: healthy eating index2015, DASH: dietary approach to stop hypertension

^a^ Body mass index (BMI) change was calculated by subtracting the BMI at baseline, from their measurements over a mean of 3 years follow-up; an increase in BMI was defined if BMI change was positive or > 0. Participants were jointly classified (8 groups), according to quartiles of healthy eating index or dietary approach to stop hypertension scores and dominant model of FTO polymorphism genotypes or genetic risk score (GRS) median ≥ and < median

^b^ Waist Hip Ratio (WHR) changes were calculated by subtracting the WHR at baseline from their measurements over a mean 3 years of follow-up; an increase in WHR was defined if their changes were positive or > 0. Participants were jointly classified (8 groups), according to quartiles of HEI or DASH scores and dominant model of FTO polymorphism genotypes or genetic risk score (GRS) median ≥ and < median

Data Are Means ± SEM (Kg/m^2^). Models were adjusted for age, sex, educational level, smoking status, physical activity, and energy intake. Q: quartiles of healthy diversity diet score, GRS: Genetic risk score

^c^ Pi: P interaction

The interaction between HEI and DASH scores by FTO SNPs (rs1121980, rs1421085, rs8050136) and GRS concerning changes in VAI and WC were shown in Table 3. Changes in WC were significantly related to FTO SNPs (rs1121980, rs1421085, rs8050136) and GRS (P trend < 0.02) across HEI score quartiles, wherein change in WC in participants in the fourth quartile of HEI in minor allele carriers of FTO rs1121980 was lower compared to the first quartile (Q1: 0.21 ± 5.49 vs. Q4: -0.03 ± 6.73, P trend = 0.03); there were similar trends regarding GRS and other SNPs. Greater adherence to DASH diet in individuals with minor allele carriers were associated with increased positive changes in WC (P trend = 0.01). There were no interactions between FTO SNPs and GRS and HEI or DASH score in relation to changes in WC or VAI.

**Table 3:** Changes in VAI and WC, according to quartiles of HEI and DASH scores by FTO genotypes and GRS in adult participants of the Tehran Lipid and Glucose Study ^a^

HEI score																	
	VAI ^a^						WC ^b^										
	Q1	Q2	Q3	Q4	p trend	pi^c^	Q1	Q2	Q3		Q4	p trend			pi^c^		
**rs1121980**						0.39									0.52		
CC	-0.01 + 0.12	-0.01 + 0.13	− 0.0.00 + 0.15	-0.02 + 0.12	0.74		0.81 + 6.29	0.90 + 5.75	0.60 + 6.31		0.11 + 5.62	0.42					
CT + TT	-0.01 + 0.07	0.00 + 0.26	-0.02 + 0.13	-0.02 + 0.13	0.34		0.21 + 5.49	1.04 + 7.55	0.59 + 6.47		-0.03 + 6.73			0.03			
**rs1421085**						0.57									0.38		
TT	-0.01 + 0.14	0.00 + 0.13	0.00 + 0.14	-0.01 + 0.10	0.55		1.25 + 6.76	0.90 + 6.16	0.80 + 6.43		0.26 + 5.37		0.25				
TC + CC	-0.01 + 0.11	-0.01 + 0.17	-0.01 + 0.14	-0.02 + 0.16	0.38		0.43 + 5.57	0.93 + 5.67	0.57 + 6.29		-0.02 + 6.02		0.03				
**rs8050136**						0.54									0.43		
GG	-0.01 + 0.14	-0.01 + 0.13	0.00 + 0.14	-0.01 + 0.11	0.64		1.18 + 6.65	0.92 + 6.16	0.65 + 6.16		0.21 + 5.45		0.36				
GA + AA	-0.01 + 0.11	-0.01 + 0.17	-0.01 + 0.14	-0.02 + 0.13	0.27		0.38 + 5.76	0.91 + 5.75	0.61 + 6.47		-0.01 + 6.07		0.02				
**GRS**						0.15									0.51		
GRS < 6.81	-0.01 + 0.13	-0.01 + 0.13	0.00 + 0.15	-0.02 + 0.12	0.72		0.10 + 5.31	1.10 + 7.21	0.67 + 6.55		-0.27 + 6.85		0.79				
GRS ≥ 6.81	-0.01 + 0.13	0.00 + 0.13	0.00 + 0.15	-0.02 + 0.12	0.35		0.84 + 6.30	0.89 + 5.76	0.62 + 6.29		0.13 + 5.61		0.02				
**DASH score**																	
	**VAI**						**WC**										
**rs1121980**						0.13									0.40		
CC	0.43 + 2.35	0.42 + 1.87	0.80 + 2.52	0.19 + 1.95	0.48		0.01 + 0.04	0.00 + 0.06		0.01 + 0.05	0.01 + 0.04	0.12					
CT + TT	0.49 + 1.99	0.37 + 1.84	0.33 + 2.43	0.34 + 1.75	0.10		0.01 + 0.05	0.01 + 0.05		0.01 + 0.05	0.02 + 0.33	0.01					
**rs1421085**						0.42									0.78		
TT	0.55 + 2.07	0.45 + 1.82	0.40 + 2.67	0.32 + 1.76	0.12		0.01 + 0.04	0.00 + 0.04		0.00 + 0.05	0.00 + 0.05		0.11				
TC + CC	0.37 + 2.02	0.28 + 1.89	0.43 + 2.00	0.36 + 1.83	0.61		0.01 + 0.06	0.01 + 0.04		0.01 + 0.04	0.04 + 0.51		0.01				
**rs8050136**						0.35									0.74		
GG	0.55 + 2.07	0.45 + 1.82	0.40 + 2.67	0.32 + 1.76	0.29		0.01 + 0.04	0.00 + 0.05		0.00 + 0.05	0.00 + 0.05		0.12				
GA + AA	0.43 + 2.01	0.31 + 1.88	0.41 + 1.93	0.34 + 1.80	0.31		0.01 + 0.05	0.01 + 0.05		0.01 + 0.05	0.04 + 0.48		0.01				
**GRS**						0.13									0.40		
GRS < 6.81	0.46 + 2.28	0.45 + 1.98	0.74 + 2.41	0.26 + 2.05	0.60		0.01 + 0.04	0.00 + 0.05		0.00 + 0.05	0.01 + 0.05		0.19				
GRS ≥ 6.81	0.49 + 1.99	0.37 + 1.81	0.33 + 2.43	0.34 + 1.75	0.15		0.01 + 0.05	0.01 + 0.05		0.01 + 0.05	0.02 + 0.33		0.01				

HEI: healthy eating index2015, DASH: dietary approach to stop hypertension, Q: quartile

^a^ A VAI (visceral adiposity index) change was calculated by subtracting the VAI at baseline, from their measurements over a mean of 3 years follow-up; an increase in VAI was defined if VAI change was positive or > 0. Participants were jointly classified (8 groups), according to quartiles of healthy eating index or dietary approach to stop hypertension scores and dominant model of FTO polymorphism genotypes or genetic risk score (GRS) median ≥ and < median

^b^ Waist circumference (WC) change was calculated by subtracting the WC at baseline, from their measurements over a mean of 3 years follow-up; an increase in WC was defined if WC change was positive or > 0. Participants were jointly classified (8 groups), according to quartiles of HEI or DASH scores and dominant model of FTO polymorphism genotypes or genetic risk score (GRS) median ≥ and < median

* ANOVA test was applied to P Trend

** ANCOVA test P interaction

Data Are Means ± SEM (Kg/m^2^). Models were adjusted for age, sex, educational level, smoking status, physical activity, and energy intake. Q: quartiles of healthy diversity diet score, GRS: Genetic risk score

^c^ Pi: P interaction

## Discussion

In the present study, no significant interactions between DASH and HEI scores and genetic predisposition in relation to changes in obesity phenotypes including BMI, WC, WHR, or VAI were found. To the best of our knowledge, few studies have investigated the interactions between HEI and DASH diets and multiple FTO genetic variants in relation to adiposity features in a population. Moreover, participants with higher GRS and minor allele carriers of FTO SNPs 1,121,980 had a lower increase in BMI and WC in the highest quartile of HEI score, compared to the lowest quartile. Adherence to the DASH diet was also associated with higher increase in WHR and WC in participants with high GRS and minor allele carriers of FTO SNPs.

Our findings are important for public health because the SNPs of FTO gene are common in our population and a high percentage of people carry these risk alleles. Moreover, Previous studies more emphasized on the interaction of certain food groups or nutrients with genetic predisposition in relation to obesity. The human diet contains numerous chemical compounds which make it difficult to investigate their separate effects on diseases; therefore, determining posteriori or priori dietary patterns and their association with obesity has been recommended. Identifying the best posteriori dietary pattern which modifies the association of FTO polymorphisms with obesity can help people adhere preventive recommendations especially in individuals with greater genetic susceptibility to obesity.

FTO is identified as a gene of interest for obesity and has been subject to many investigations, most of which confirming that there is a notable association between FTO polymorphism and obesity traits [26, 27]. Studies targeting certain FTO SNPs have reported higher BMI scores in risk allele carriers of European descent [28, 29]. Moreover, common variation in FTO gene was seen to be associated with increased BMI in a large meta-analysis studying Chinese population [30]. Another large-scale meta-analysis showed that the homozygous FTO risk allele was associated with a 23% higher risk of obesity [31]. Studies conducted in Iran showed similar results [25]; in a cross-sectional study of 198 participants, it was shown that homozygous carriers of the risk allele of FTO rs9939609 had higher values for BMI, WHR, WC, and fat mass [32]. Though the exact mechanism underlying this phenomenon has not yet been established, it was proposed that the FTO gene could regulate fat distribution, satiety, and energy intake [33]. Moreover, there are evidences suggesting that certain variants of the FTO gene are associated with insulin resistance and higher inflammatory response [34, 35].

Previous studies showed that SIRT1 potentially prevented excessive accumulation of fat in adipose tissue. The interesting point is that recent studies have shown that sirtuins exert their role in energy metabolism in response to nutrient signals. Giving resveratrol to rats fed a high-fat diet protected them from high-fat-induced obesity, and this protective effect was due to increased activation of sirtuins by resveratrol [36, 37]. Therefore, it is possible that the genetic expression of sirtuins as a factor affected by the diet is effective on the results of our study; because the expression of the sirtuins gene may be affected by the HEI and DASH diet thereby cover the effects of FTO polymorphism on the changes in obesity traits.

Investigating a whole dietary pattern could provide a better insight into the progression of a certain condition, compared to evaluating the effects of a single nutrient or food [38]. It is well established that the DASH diet is the preferable approach to treat and control high blood pressure. Although the effect of DASH diet on hypertension is believed to be beneficial regardless of its impact on weight [39], several clinical studies have reported more significant weight loss when DASH and low-calorie diets are combined [40]. Moreover, a recent systematic review and meta-analysis of 13 trial studies revealed that adherence to the DASH diet could contribute to weight loss and decreased BMI and WC regardless of energy intake, further strengthening the notion of a possible role of the DASH diet in weight reduction [41]. Like the DASH diet, HEI was not specifically designed for weight management; however, poor diet, as defined by lower HEI scores had been indicated to induce obesity [42]. HEI has also been indicated as a good predictor of future obesity in adulthood [43]. Additionally, there were a lot of observational studies attributing that higher adherence to HEI predicted lower obesity risk [44, 45].

In the current study, lower adherence to HEI in minor allele carriers of FTO variants led to a higher increase in BMI and WC. Moreover, individuals in the highest quartile of the DASH diet had a lower increase in WHR and WC compared to the individuals in the lowest quartile. However, no significant interaction was seen between FTO polymorphisms and DASH and HEI diets regarding changes in obesity indices. Recent studies regarding the gene-diet interaction reported controversial findings; in line with our results, a recent meta-analysis did not detect any interactions between protein intake and genetic predisposition to obesity on BMI, WC, or WHR [46]. Livingstone et al. found no significant interaction between the Mediterranean Diet, HEI and FTO polymorphism concerning obesity changes [47]. Two other studies reported the same results [48, 49]. A study led by de Luis et al. revealed that a 3-month hypocaloric low-fat dietary intervention among different variants of rs9939609 polymorphisms carriers led to bodyweight decrease in both genetic groups independently of allele T or A carriage [50].

On the other hand, there are a number of studies in which significant diet-gene interplay had been discovered. A recent study reported a notable interaction between GRS and intakes of energy, protein, total fat, SFA, poly-unsaturated fat (PUFA), and carbohydrate on BMI, body fat mass, and WC [51]. A significant interaction between sugar-sweetened beverages and genetic predisposition to obesity was seen in a review of 3 large cohort studies [52]. In an Asian Indian population, carbohydrate and fiber intake modulated the association of FTO SNPs rs8050136 and rs11076023 with obesity traits [53]. Findings from several clinical trials conducted on the matter suggested a significant role of the genotypes of FTO influencing weight loss after lifestyle interventions [54, 55]. The inconsistency of the current evidence regarding the gene-diet interplay emphasizes the need for further research in this area.

Results of the present study on DASH score and WC/ WHR in individuals with minor allele of FTO variants are opposite of the reports of previous studies, which suggested that high adherence to DASH diet is associated with reduction in the risk of abdominal obesity [56]. This controversy may be due to that the mean DASH score observed in our study was around 30.0 for the upper quartile, it is possible that the mean DASH score should be higher than 30 in order to exert its protective effects on abdominal obesity or it is possible that the mean of each food group intakes in each quintile of our study was inconsistent with other studies, Moreover subjects who are in the highest quintile of dietary intake for each food group might not meet the current recommendations of that food group.

Different findings might also be explained by the different definition of the DASH diet among studies. For example, in our study, nuts and legumes were in the same group and they are scored together. Also DASH dietary pattern depends on energy intake reduction to exert protective effects on abdominal obesity management and if this reduction in caloric intake does not occur, abdominal obesity may increase, so DASH score was not a good choice for weight management in minor allele (risk allele) carriers of rs1121980, rs1421085 and rs8050136.

The strengths of our study include its prospective design and utilizing a pre-defined dietary pattern analysis to better investigate the effect of overall dietary composition. Detailed information on physical activity, BMI, smoking status allowed extensive adjustment for obesity risk factors.

### Limitations

There are some limitations that should be noted; our study population was highly homogenous as the subjects reside roughly in the same part of the town. These findings should be determined in other race and ethnicities. Moreover, there might be other potential confounders like economical influence and other social factors that could not be measured or adjusted. Our study included only three SNPs of the FTO gene whereas there are certainly more polymorphisms of FTO and other genes like MC4R, OLFM4, TCF7L2, ADCY3, GNPDA2, MAP2K5, and NRXN3 to be investigated. Besides, despite FFQ benefits, is not a robust tool to measure an individual’s exact dietary assessment. The major limitation of our study is calculating the DASH score, as the quintile levels of each food group was based on individuals’ dietary intake, so the range of each food group intake in each quintile of our study may be inconsistent with other studies. Finally, in order to have more conclusive findings, further studies with longer follow-up period are warranted.

## Conclusion

Our study revealed that there was no notable interaction between adherence to DASH diet or HEI and genetic predisposition on the obesity indices. However, adherence to HEI and DASH diets modified the association between FTO genetic variations and obesity phenotypes. In minor allele (risk allele) carriers of FTO polymorphisms, low change in BMI and WC were seen with high adherence to the HEI. Conversely, high adherence to the DASH diet by this genotypic group was related to increasing WC.

### Electronic supplementary material

Below is the link to the electronic supplementary material.

**Supplementary Table 1.** STROBE-nut: An extension of the STROBE statement for nutritional epidemiology

## Data Availability

The datasets used and/or analyzed during the current study are available from the corresponding author on reasonable request.

## Abbreviations

BMI: Body mass index
CVD: Cardiovascular diseases
DASH: Dietary approach to stop hypertension
FFQ: Food frequency questionnaire
FTO: Fat mass and obesity associated gene
GRS: Genetic risk score
HEI: Healthy eating index
MET: Metabolic equivalent
SNP: Single nucleotide polymorphisms
SFA: Saturated fat
TLGS: Tehran Lipid and Glucose Study
VAI: Visceral adiposity index
WC: Waist circumference
WHR: Waist to hip ratio
